# Commentary: Causal effects of specific gut microbiota on bone mineral density: a two-sample Mendelian randomization study

**DOI:** 10.3389/fendo.2024.1346562

**Published:** 2024-02-28

**Authors:** Jiahao Chen, Xiang Guo, Hong Song

**Affiliations:** School of Basic Medicine, Zhejiang Chinese Medical University, Hangzhou, Zhejiang, China

**Keywords:** gut microbiota, bone mineral density, Mendelian randomization, Prevotella, allele

## 1 Introduction

Shuai Chen and his colleagues published a study titled “Causal effects of specific gut microbiota on bone mineral density: a two-sample Mendelian randomization study” in *Frontiers in Endocrinology* (1). The authors concluded, “Our MR analysis also found that the abundance of Prevotella9 and Prevotellaceae was associated with high BMD at different sites. Consistent directional effects for all analyses were observed in both MR-Egger and weighted median methods, which suggests that Prevotella might be a promising target for osteoporosis prevention.” We support and appreciate the authors’ work and agree with their conclusions. However, we have some concerns about their data and results. Here are some critical comments on these issues.

On the first line of Supplementary Table S1, the Effect allele and Other alleles were marked wrong. In the article of Qi Wang et al. (2–4), the Effect_allele of rs182549 was C, and Other_allele was T, while in the author’s article, the Effect_allele of rs182549 was T, and Other_allele was C. In short, “Effect_allele” and “Other_allele” should switch places in the Supplementary Table S1.

Under the subheading of Causal effect of the gut microbiota on forearm bone mineral density, this article first describes “Genus Prevotella9 (β = 0.129, 95% CI: 0.007–0.251, P = 0.039) were negatively associated with FA-BMD (Figure 2)”. Then, in *Discussion*, this article describes “which suggests that Prevotella might be a promising target for osteoporosis prevention.” There is an apparent inconsistency between the two accounts. β is the beta value, and exposure factors and outcomes are positively correlated when β is >0 (5–7). Hence, Prevotella9 was positively correlated with FA-BMD, which is consistent with the study of Liang Zhuang et al., “Some short-chain fatty acids (SCFAs) producers, including Lactobacillus, Akkermansia, Prevotella, Alistipes, and Butyricicoccus, were reduced in patients with lower bone mass (LBM) and PMO.” (8).

The study by Chen and his colleagues has helped us better understand the relationship between specific gut microbiota and bone mineral density. Still, it needs to be written more accurately to demonstrate the causal relationship between the gut microbiota and the risk of osteoporosis.

## Author contributions

JC: Writing – original draft, Investigation. XG: Investigation, Writing – original draft. HS: Writing – review & editing.
